# Combined apophyseal and epiphyseal fixation of Ogden type IIIA/IV tibial tubercle avulsion fractures provides favorable stability compared to isolated apophyseal screw fixation - a biomechanical study

**DOI:** 10.1007/s00068-025-02814-w

**Published:** 2025-03-18

**Authors:** Christian Peez, Ivan Zderic, R. Geoff Richards, Ludmil Drenchev, Hristo K. Skulev, Boyko Gueorguiev, Christoph Kittl, Michael J. Raschke, Elmar Herbst

**Affiliations:** 1 Department for Trauma, Hand and Reconstructive Surgery, University Hospital Münster, Münster, Germany; 2 AO Research Institute, Davos, Switzerland; 3 Bulgarian Academy of Sciences, Institute of Metal Science “Acad. A. Balevski”, Sofia, Bulgaria

**Keywords:** Tibial tubercle avulsion fracture, Ogden fracture, Fracture type-specific fixation, Biomechanics

## Abstract

**Purpose:**

Current literature lacks recommendations regarding proper fixation of tibial tubercle avulsion fractures involving the proximal tibial epiphysis (Ogden fractures). Therefore, the aim of this study was to compare isolated apophyseal screw fixation and additional fixation techniques in Ogden fractures.

**Methods:**

Two different types of apoepiphyseal tibial tubercle avulsion fractures were created in 40 proximal tibiae according to the modified Ogden classification: (1) Ogden type IIIA and (2) Ogden type IV. The fractures were fixed with either isolated apophyseal screws or additionally with a medial plate or epiphyseal screws. All specimens were biomechanically tested under progressively increasing cyclic loading until failure, while capturing the interfragmentary movements with motion tracking.

**Results:**

Augmentation of apophyseal screw osteosynthesis by a medial plate in Ogden IV fractures or epiphyseal screws in Ogden IIIA fractures exhibited significantly higher cycles to failure and failure loads (*P*< 0.05), and significantly less axial displacement (*P* < 0.05) compared to isolated apophyseal screw fixation. Fixation of Ogden type IIIA fractures resulted in significantly less axial displacements and higher construct stiffness, cycles to failure and failure loads compared to Ogden type IV fracture (*P* < 0.001). Fracture gap opening did not differ significantly between the fixation techniques.

**Conclusions:**

Augmented apophyseal screw fixation of apoepiphyseal tibial tubercle avulsion fractures provides greater biomechanical stability than isolated apophyseal screw fixation. Regardless of fixation technique, Ogden type IV fractures are more unstable than Ogden type IIIA fractures, so an individualized treatment strategy based on fracture morphology is crucial. In case of an Ogden type IIIA or Ogden type IV fracture, surgeons should consider adding epiphyseal screws or a medial plate osteosynthesis to apophyseal screw fixation to best neutralize forces of the extensor mechanism, as long as the often compromised soft tissue envelope can tolerate greater surgical invasiveness.

## Introduction

Tibial tubercle avulsion fractures represent a rare but severe injury to the knee joint in adolescence accounting for 0.4–2.7% of pediatric fractures and 1% of all physeal injuries, with an increasing incidence in male athletes [1–4]. Despite the relatively high complication rate of 28% [3, 5], functional and radiographic outcomes following surgical treatment have been reported to be excellent, with high rates of fracture union and return to preinjury activity levels [3, 4, 6–9]. However, these fractures frequently present with proximal epiphyseal involvement (Ogden type III/IV), with more than 50% being intra-articular injuries [5, 10]. In fact, these intra-articular injuries are associated with poorer functional outcomes, suggesting that current management could be optimized [3, 9]. Key factors in improving patient-reported outcomes are the restoration of the extensor mechanism and an anatomic reconstruction of the articular surface [11–14], for which a proper fracture reduction and fixation are essential [10, 15–18].

Irrespective of the fracture type, various fixation techniques have been proposed to achieve absolute stability and interfragmentary compression of the avulsed tibial tubercle [4], with apophyseal screw fixation representing the most common internal fixation method [4, 10, 19–21]. However, this fixation technique differs not only in the number, configuration and design of the screws, but also in additional stabilization with tension bands, cerclage, periosteal sutures, suture anchors or plates [4]. During normal gait, the extensor mechanism and therefore the osteosynthesis are exposed to high moments and forces, so that especially in combined tibial tubercle and epiphyseal injuries an additional stabilization of the apophyseal screws may be required to neutralize these extension forces [22–24]. However, the biomechanical stability of different techniques for augmentation of apophyseal screw osteosynthesis in Ogden type III/IV fractures has not yet been discussed, so that recommendations regarding proper fixation of these apoepiphyseal injuries are lacking.

Therefore, the aim of this study was to compare the biomechanical competence of different augmented constructs and isolated apophyseal screw fixation in Ogden type III/IV fractures. It was hypothesized that (1) augmented apophyseal screw fixation would demonstrate favorable stability compared to an isolated apophyseal screw fixation and (2) the stability of the fixation technique depends on the Ogden fracture type.

## Materials and methods

### Specimens and preparation

Forty fresh-frozen (-20 °C) non-paired human cadaveric knees from 20 female and 20 male donors aged 76.0 ± 7.3 years (mean ± standard deviation, SD) (range 61–88 years) were obtained from an international tissue bank (Science Care, Phoenix, Arizona, United States of America). Institutional Review Board approval was obtained from the AO Research Institute Davos to conduct this biomechanical study (PP2115, 6 February 2018). The donors gave informed consent to the use of their corpse in medical science during their lifetime, so that the specimens were dissected and biomechanically tested in accordance with the relevant guidelines and regulations.

Bone mineral density (BMD) within the trabecular region of the proximal tibial metaphysis was assessed in each specimen using computed tomography (CT) scanning (Revolution EVO, General Electric Healthcare, Buckinghamshire, UK). A phantom (BDC-6, QRM GmbH, Möhrendorf, Germany) was subsequently analyzed using an image processing software (Amira, v.6.0, Thermo Fisher Scientific, Waltham, MA, USA) with segmentation between 150 and 450 mgHA/cm^3^.

Based on BMD, the knees were assigned to two clusters consisting of 24 and 16 specimens, with a homogenous BMD distribution between the clusters (n.s.). Within each cluster, a different type of apoepiphyseal tibial tubercle avulsion fracture was simulated according to the modified Ogden classification [13, 25]: (1) Type IIIA fractures, tibial tubercle avulsion fractures with a coronal plane fracture extending proximally into the tibial plateau (*n* = 24); and (2) Type IV fractures, tibial tubercle avulsion fractures through the proximal tibial physis and avulsion of the entire tibial plateau (*n* = 16).

Prior to preparation and biomechanical testing, the knees were thawed at room temperature for 24 h. The tibia and fibula were then cut 250 mm distal to the knee joint line, and the knee joints were disarticulated to harvest the proximal lower leg. The entire soft tissue was dissected while preserving the interosseous membrane and articular capsule of the proximal tibiofibular joint. Once the patella was removed and the fibula was secured to the tibia in its anatomical position with a 3.5 mm tri-cortical position screw, the specimens were wrapped in phosphate buffered saline soaked tissue paper to prevent tissue dehydration.

**Fig. 1:**
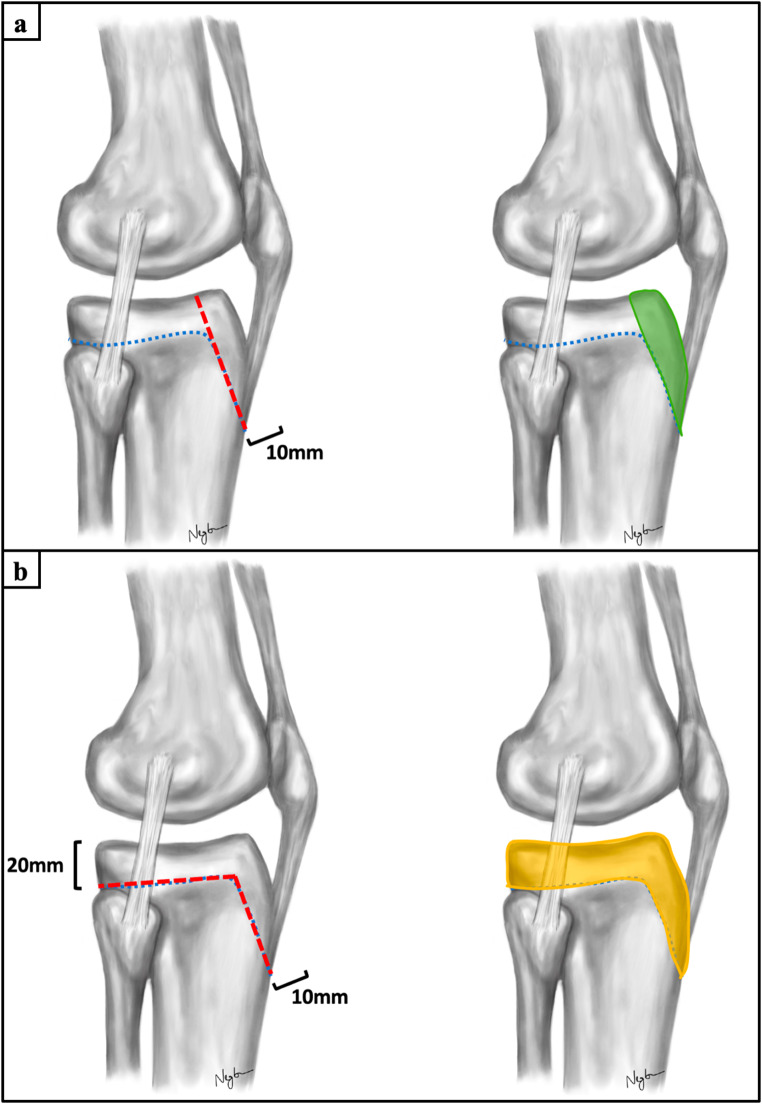
Schematic illustration of the fracture models. An Ogden type IIIA fragment (**a**, green and an Ogden type IV fragment (**b**, orange) were created to simulate apoepiphyseal tibial tubercle avulsion fractures

Based on the cluster assignment, Ogden type IIIA and Ogden type IV fractures were simulated by osteotomies using custom-made cutting guides attached to the medial surface of the proximal tibia [13, 25]. To mimic an Ogden type IIIA fracture, a coronal plane osteotomy of the tibial tuberosity was performed to a fragment thickness of 10 mm. The osteotomy started immediately distal to the tibial attachment of the patellar tendon and extended proximally into the tibial plateau through the tibial attachment site of the anterior cruciate ligament to create a tibial tubercle avulsion fracture with a coronal split fracture of the entire tibial plateau (Fig. 1a). To reproduce a Ogden type IV fracture, the epiphysis was marked medially with two 2.0 mm Kirschner (K-) wires 20 mm distal to the medial tibial plateau corresponding to the average epiphyseal height of the proximal tibial in adolescents [26]. Then, a coronal plane osteotomy of the tibial tuberosity was performed to a fragment thickness of 10 mm, starting immediately distal to the tibial attachment of the patellar tendon. Finally, an axial plane osteotomy parallel to the tibial plateau along the marked physis completed the creation of a tibial tubercle avulsion fracture with avulsion of the entire tibial plateau (Fig. 1b).

**Fig. 2:**
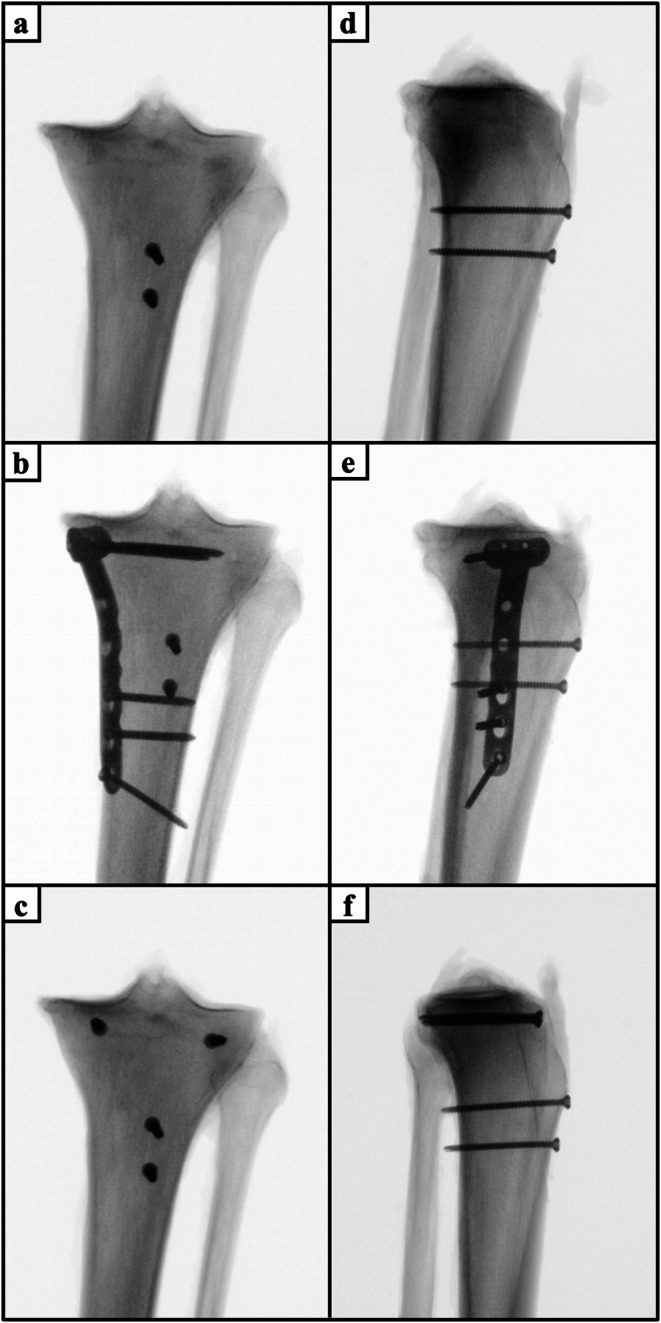
Anteroposterior (**a-c**) and mediolateral (**d-f**) radiographs of specimens with Ogden type IIIA tibial tubercle avulsion fractures treated by isolated apophyseal screw fixation (**a**, **d**) as well as additional medial plate fixation (**b**, **e**) or epiphyseal screw fixation (**c**, **f**)

**Fig. 3:**
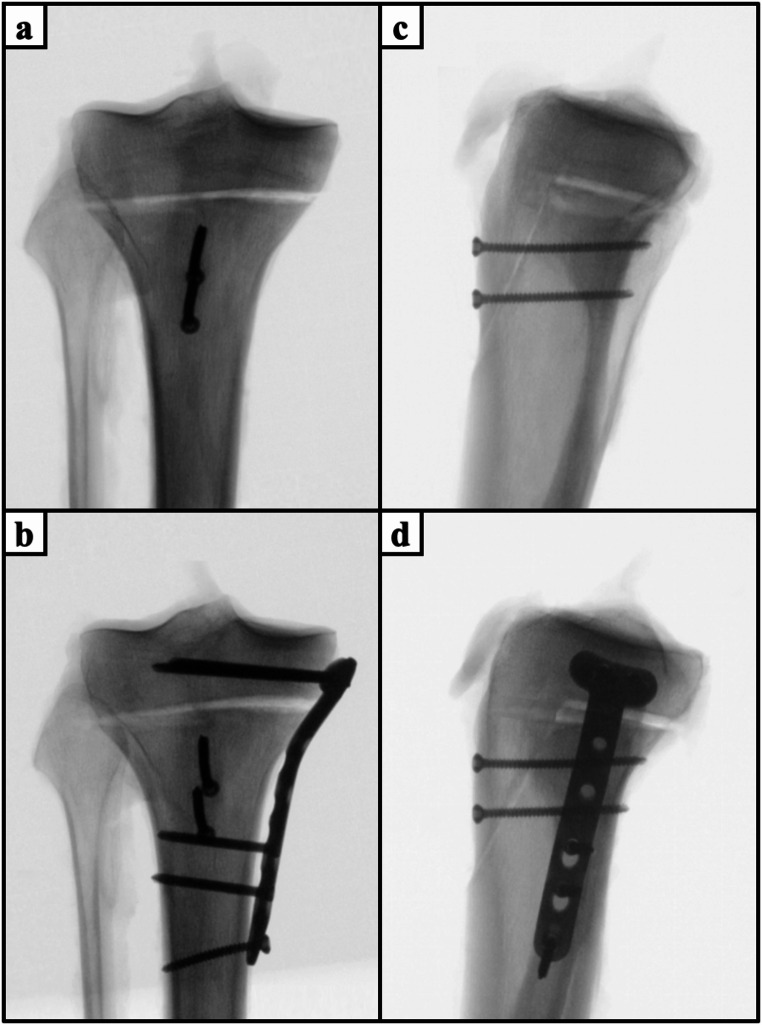
Anteroposterior (**a**, **b**) and mediolateral (**c**, **d**) radiographs of specimens with Ogden type IV tibial tubercle avulsion fractures treated by isolated apophyseal screw fixation (**a**, **c**) and additional medial plate fixation (**b**, **d**)

Depending on the specimen’s group assignment, the Ogden IIIA fractures were treated with either isolated apophyseal screw fixation (Fig. 2a and d) or additionally with either medial plate fixation (Fig. 2b and e), or epiphyseal screw fixation (Fig. 2a and f). Complementary, the Ogden IV fractures were treated according to the specimen’s group assignment with either apophyseal screw fixation (Fig. 3a and c) or additionally with medial plate fixation (Fig. 3b and d). For apophyseal screw fixation, two 3.5mm fully-threaded cortical screws (Johnson&Johnson MedTech, Zuchwil, Switzerland) were inserted perpendicular to the coronal plane fracture of the tibial tuberosity and anchored bicortically to the posterior tibial cortex. Additional medial plate fixation was performed using a 3.5 mm 3-hole locking compression plate (LCP Medial Proximal Tibia Plate, Johnson&Johnson MedTech, Zuchwil, Switzerland). The epiphyseal segment was secured with three 3.5 mm unicortical self-tapping locking screws, while the diaphyseal segment was fixed with three 3.5 mm self-tapping cortical screws. For additional epiphyseal screw fixation, a 3.5mm fully-threaded cortical screw (Johnson&Johnson MedTech, Zuchwil, Switzerland) was inserted into the epiphyseal fragment subchondral to the medial and lateral tibial plateau and anchored bicortically to the posterior tibial cortex. The screw trajectories were perpendicular to the coronal plane fracture of the tibial tuberosity and parallel to the tibial plateau.

Upon completion of the surgical procedures, the distal 6 cm of the proximal tibiae were embedded in a polymethylmethacrylate (PMMA, Suter Kunststoffe AG, Fraubrunnen, Switzerland) socket. Finally, retro-reflective maker sets were attached to the tibial shaft and Ogden fragment for motion tracking.

### Biomechanical testing

**Fig. 4:**
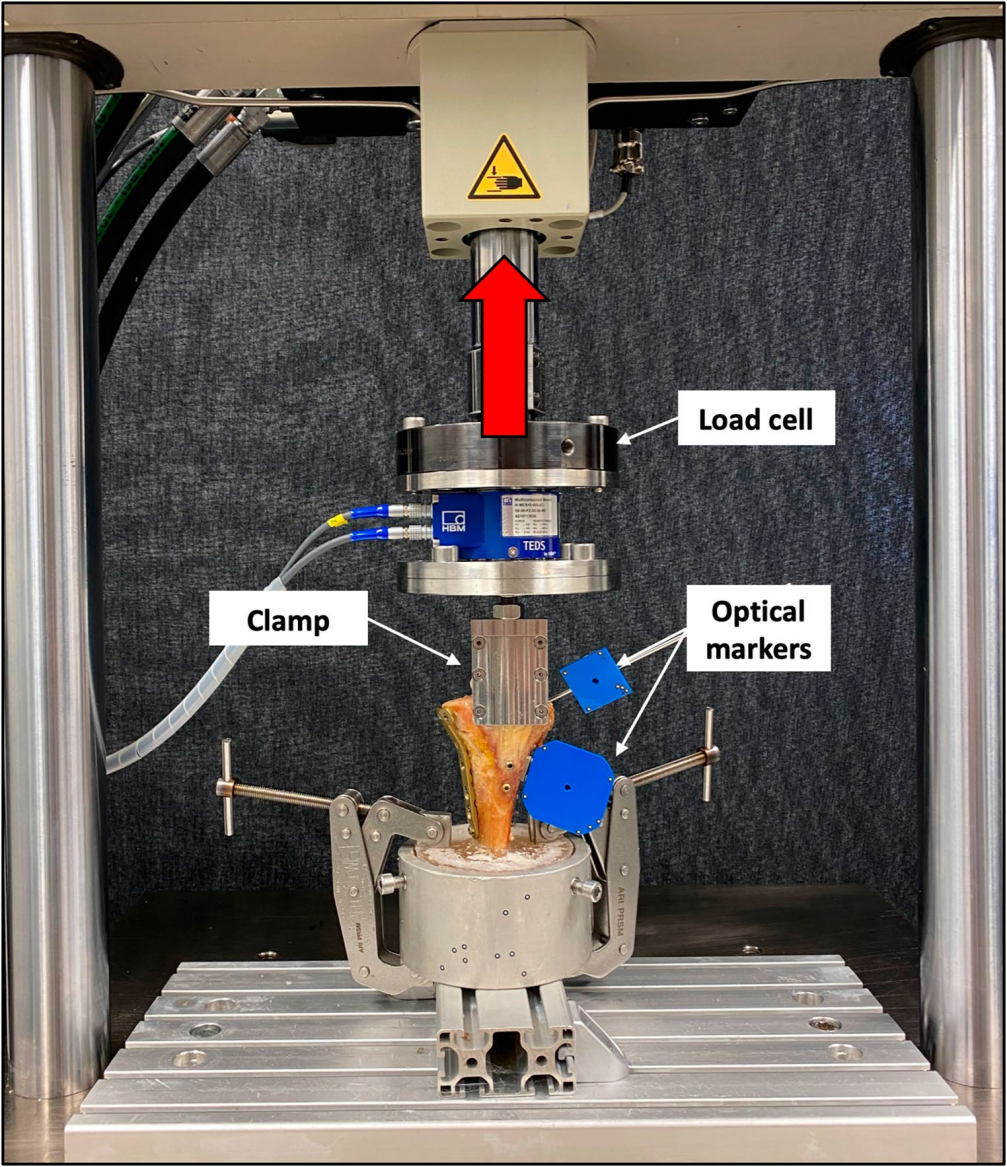
Test setup with a specimen mounted for biomechanical testing. The vertical arrow denote loading direction for tensile loading directed via a clamp to the patellar tendon

Biomechanical testing was performed using a servo-hydraulic materials testing machine (Bionix 858.20, MTS Systems Corp., Eden Prairie, MN, USA) equipped with a 5 kN load cell (HBM, Darmstadt, Germany), that allows position and force control with 0.05% accuracy. Each specimen was tested in an upright standing position, with the distal tibial PMMA socket rigidly mounted to the machine base plate. Pulling force was transmitted to the patellar tendon via a custom-made steel clamp attached to the machine actuator, leaving 20 mm of free tendon between the clamp and the tibial tubercle. The orientation of the patellar tendon and the force vector were perpendicular to the joint line of the proximal tibia, corresponding to a worst-case scenario of load applied to the extensor mechanism (Fig. 4).

Starting from a preload of 20 N, axial tensile loading commenced with a non-destructive quasi-static ramp to 50 N at a rate of 10 N/sec, followed by a progressively increasing cyclic loading at 2 Hz. While maintaining a constant valley load of 20 N, the peak load increased monotonically from 50 N at a rate of 0.025 N/cycle until reaching the limit of 10 mm actuator displacement.

### Data acquisition and evaluation

Actuator displacement and axial pulling forces were recorded by the test system controllers of the testing machine at 128 Hz. Based on these data, force-displacement curves were generated to calculate the construct stiffness, defined as the slope of the initial quasi-static ramp within the linear loading range between 20 and 50 N.

In addition, a stereographic optical motion tracking system using contactless full-field deformation technology (Aramis SRX, Carl Zeiss GOM Metrology GmbH, Braunschweig, Germany) continuously captured the coordinates of the attached optical markers in all six degrees of freedom, operating at a maximum acceptance error of 0.004 mm [27]. Based on these, interfragmentary movements were evaluated at the initial stage and thereafter at 1000, 2000, 3000, 4000 and 5000 cycles under peak loading conditions with respect to the beginning of the cyclic test. Specifically, fracture site displacement along the tibial shaft axis, defined as axial displacement, was captured between the most distal margin of the Ogden fragment at the tibial tubercle and the tibial shaft. Further, interfragmentary rotation around the mediolateral axis, defined as fracture gap opening, of the Ogden fragment with respect to the tibial shaft axis was evaluated. Fracture step-offs greater than 2 mm are associated with increased articular pressures [28, 29] and therefore early onset of osteoarthritis [30–33] following tibial plateau fractures. As Ogden IIIA fractures represent an intra-articular injury with involvement of the tibial plateau, reaching an axial displacement of 2 mm was set as the clinically relevant failure criterion. The number of cycles until its fulfillment under peak loading were defined as cycles to failure, followed by a calculation of the corresponding failure load.

### Statistical analysis

Statistical analysis was performed using Prism (Version 9, GraphPad Software, Boston, USA). Descriptive data is presented as mean values ± SD. Normality of data distribution within each fixation technique was tested and proved using the Shapiro-Wilk test. Significant differences among the groups regarding BMD, construct stiffness, cycles to failure and failure load were detected by a One-Way Analysis of Variance with Geisser-Greenhouse correction and Tukey´s post hoc test for multiple comparison. Two-Way Repeated-Measures ANOVAs with Geisser-Greenhouse correction and post hoc Sidak´s multiple comparison test were conducted to identify significant differences among the groups with regard to axial displacement and fracture gap opening evaluated over 5000 test cycles. Overall level of significance was set at *P* = 0.05.

An a-priori power analysis was performed using G*Power-2 software (University Düsseldorf, Düsseldorf, Germany) [34]. Based on mean values and standard deviations from previous studies evaluating the biomechanical performance of different screw designs in tibial tubercle osteotomies [35, 36], it was assumed that a sample size of 8 would allow the identification of changes in displacement of 3.0 mm with a SD of 2.0 mm (effect size / Cohen`s d = 1.5) with 80% power, at the significance level of *P* < 0.05.

## Results

The BMD (mgHA/cm^3^) ranged from 124.4 ± 39.6 to 131.5 ± 30.3, demonstrating a homogenous distribution among the groups (*P* > 0.997) (Table 1).

**Table 1:** Outcome measures for the investigated parameters of interest bone mineral density, construct stiffness, cycles to failure and corresponding failure loads, shown for each fixation technique/group separately in terms of mean value and standard deviation, together with the corresponding p-value from the statistical comparison of the augmented constructs versus isolated apophyseal screw fixation. N.a. = not applicable

Parameters	Ogden type IIIA fracture		
	Apophyseal screws	Apophyseal screws + Medial Plate	Apophyseal screws + Epiphyseal screws
**Bone Mineral Density [mgHA/cm** ^**3**^ **]**	124.4 ± 39.6	126.5 ± 39.1 (0.997)	130.5 ± 42.9 (> 0.999)
**Construct stiffness [N/mm]**	237.4 ± 104.2	238.7 ± 69.8 (0.998)	219.5 ± 47.4 (0.768)
**Cycles to failure**	3713.0 ± 1174.5	10130.1 ± 2912.4 (**< 0.001**)	8979.8 ± 2633.5 (**< 0.001**)
**Failure load** **[N]**	148.08 ± 34.1	304.7 ± 86.0 (**< 0.001**)	296.3 ± 84.4 (**< 0.001**)
**Parameters**	**Ogden type IV fracture**		
	**Apophyseal screws**	**Apophyseal screws** **+ Medial Plate**	**Apophyseal screws** **+ Epiphyseal screws**
**Bone Mineral Density [mgHA/cm** ^**3**^ **]**	125.8 ± 25.0	131.5 ± 30.1 (0.997)	n.a.
**Construct stiffness [N/mm]**	81.9 ± 24.5	89.5 ± 46.0 (0.952)	n.a.
**Cycles to failure**	2776.6 ± 1513.0	4843.9 ± 1263.9 (**< 0.05**)	n.a.
**Failure load** **[N]**	127.1 ± 38.7	158.4 ± 34.4 (**< 0.05**)	n.a.

### Ogden type IIIA fracture

**Fig. 5:**
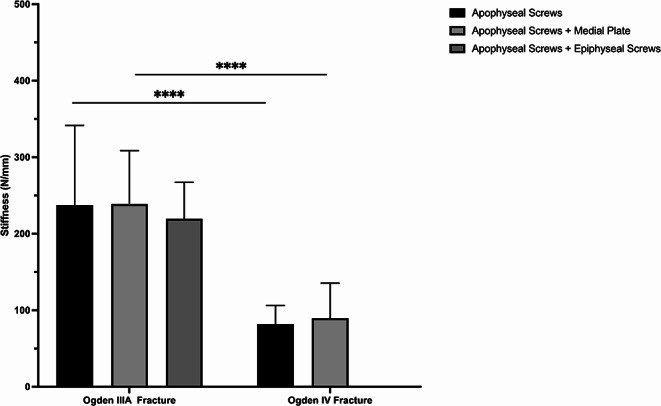
Construct stiffness following fixation of Ogden type IIIA/IV tibial tubercle avulsion fractures with either isolated apophyseal screws or additionally with medial plate fixation or epiphyseal screw fixation. Error bars indicate mean value ± standard deviation. **** = *P* < 0.0001

**Fig. 6:**
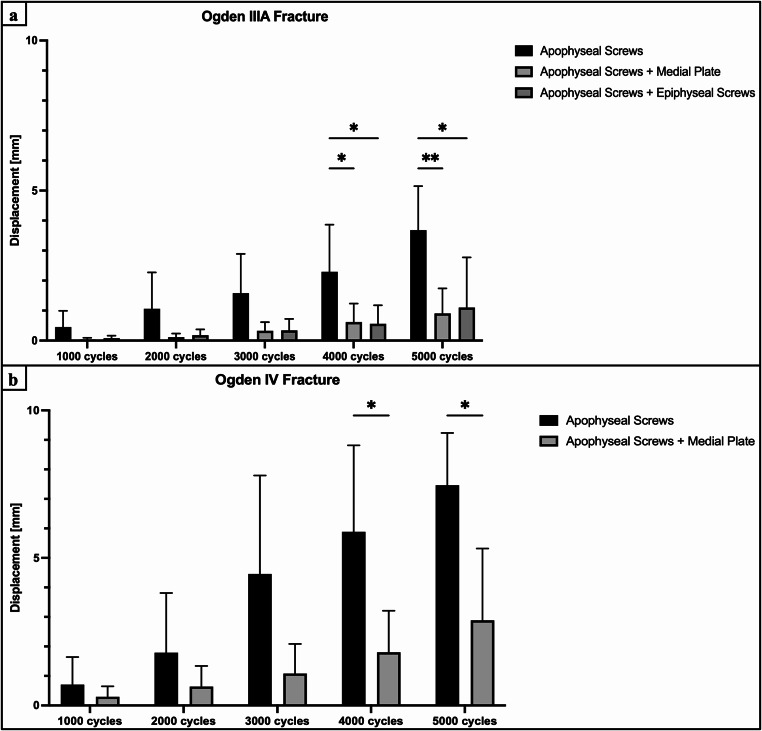
Axial displacement after 1000, 2000, 3000, 4000, and 5000 cycles, shown separately for each group as mean value and standard deviation, together with the corresponding p-value from the statistical comparisons between groups. (**a**) Ogden type IIIA tibial tubercle avulsion fracture. (**b**) Ogden type IV tibial tubercle avulsion fracture. * = *P* < 0.05, ** = *P* < 0.01

**Fig. 7:**
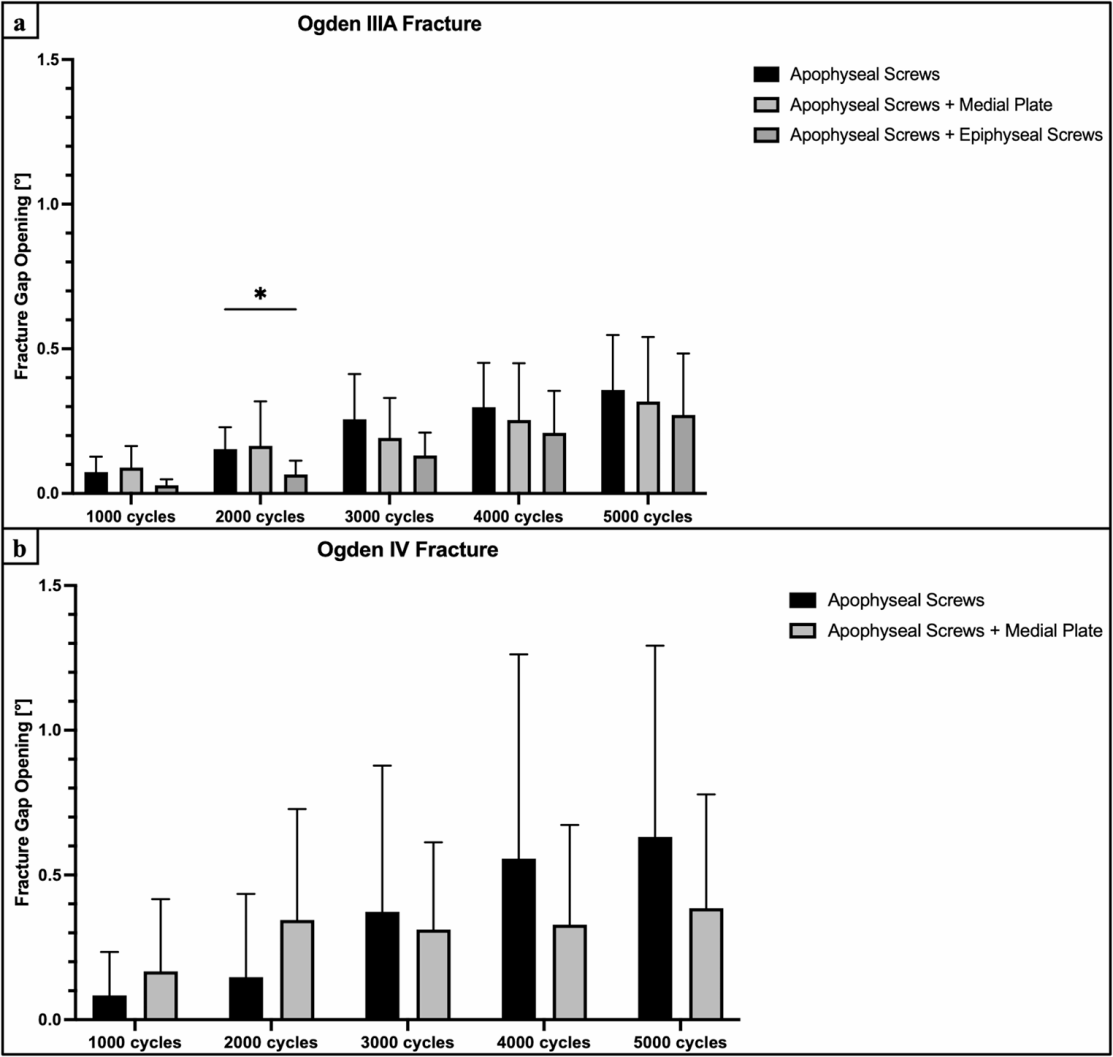
Fracture gap opening after 1000, 2000, 3000, 4000, and 5000 cycles, shown separately for each group as mean value and standard deviation, together with the corresponding p-value from the statistical comparisons between groups. (**a**) Ogden type IIIA tibial tubercle avulsion fracture. (**b**) Ogden type IV tibial tubercle avulsion fracture. * = *P* < 0.05

**Fig. 8:**
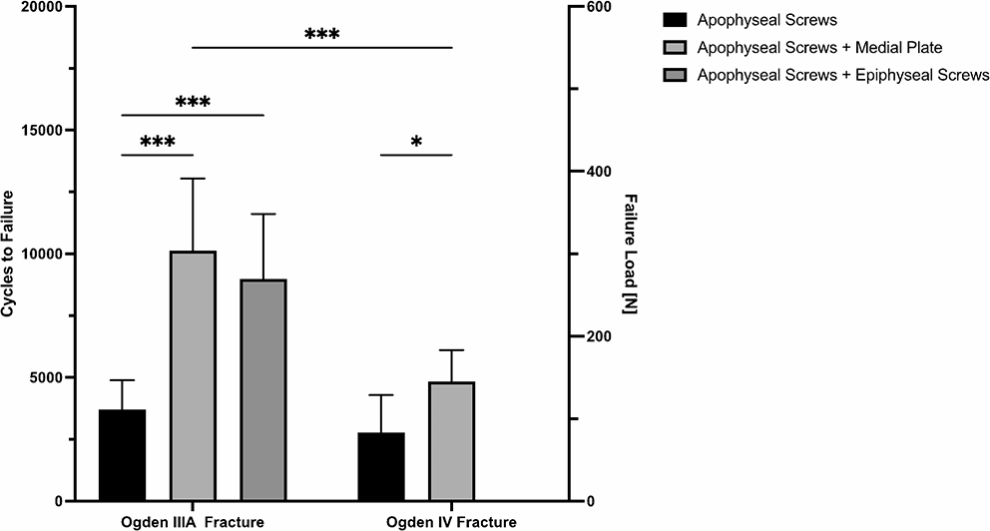
Cycles to failure and corresponding failure load following fixation of Ogden type IIIA and Ogden type IV tibial tubercle avulsion fractures with either isolated apophyseal screws or additionally with medial plate fixation or epiphyseal screws. Error bars indicate mean value ± standard deviation. * = *P* < 0.05, *** = *P* < 0.001

Construct stiffness of additional medial plate fixation and epiphyseal screw fixation was comparable to isolated apophyseal screw fixation (*P* > 0.768) (Fig. 5). Medial plate-augmented and epiphyseal screw-augmented fixation of the Ogden IIIA fragment exhibited significantly less axial displacement after 4000 cycles compared to isolated apophyseal screw fixation (*P* < 0.05) (Fig. 6a). During the 5000 cycles, fracture gap opening was not significantly different between these three techniques (*P* > 0.118), except for the combined apoepiphyseal screw fixation at 2000 cycles (*P* < 0.05) (Fig. 7a). Cycles to failure and corresponding failure loads were not significantly different between the two augmented apophyseal screw fixations (*P* > 0.798), while isolated apophyseal screw fixation resulted in earlier construct failure with significantly less cycles to failure and failure loads (*P* < 0.001) (Fig. 8; Table 1).

### Ogden type IV fracture

Construct stiffness of additional medial plate fixation was comparable to isolated apophyseal screw fixation (*P* > 0.952) (Fig. 5). Augmented medial plate constructs exhibited significantly less axial displacement of the Ogden IV fragment after 4000 cycles compared to isolated apophyseal screw fixation (*P* < 0.05) (Fig. 6b), whereas fracture gap opening did not significantly differ between these two fixations over 5000 cycles (*P* > 0.817) (Fig. 7b). Additional medial plate fixation of the Ogden IV fragment provided higher cycles to failure and failure loads compared versus isolated apophyseal screw fixation (*P* < 0.05) (Fig. 8; Table 1).

### Effect of fracture type

Surgically treated Ogden type IIIA fractures with isolated apophyseal screw fixation and augmented medial plate fixation exhibited significantly less axial displacement after 5000 cycles and significantly higher construct stiffness, cycles to failure and failure loads compared to Ogden type IV fracture treated with the same technique (*P* < 0.001). Over 5000 cycles, fracture gap opening of isolated apophyseal screw fixation and additional medial plate fixation in Ogden type IIIA was comparable to Ogden type IV fractures (*P* > 0.556).

## Discussion

The most important finding of the present study was that augmented apophyseal screw fixation of Ogden type IIIA and Ogden IV fractures provided higher biomechanical stability than isolated apophyseal screw fixation, with less fracture site displacement as well as a higher number of cycles to failure and corresponding failure loads. However, isolated and augmented apophyseal screw fixation of Ogden type IV fractures resulted in larger fracture site displacement and earlier construct failure with less cycles to failure and failure loads than surgically treated Ogden type IIIA fractures, suggesting that an individualized treatment strategy based on fracture morphology is crucial.

Even though surgical treatment of tibial tubercle avulsion fractures is frequently associated with excellent functional and radiographic outcomes [3, 4, 6–9, 20, 21, 37], quadriceps atrophy and extension strength deficits are common after extensor mechanism injuries [9, 38, 39]. In fact, Riccio et al. [9] reported poorer extension torque generation in surgically treated Ogden fractures at 3-years follow-up, with 26% of subjects revealing a minimal clinical important difference in peak quadriceps torque of more than 20% and on average 31% less quadriceps strength compared to the uninjured limb. In addition, tibial tubercle avulsion fractures with proximal epiphyseal involvement and/or intra-articular extension (Ogden type III/IV) tended to have poorer functional and patient-reported outcomes, with reduced range of motion and fewer patients returning to preinjury levels of activity [3, 4, 9]. Besides neuromuscular deficits and injury-related cartilage and meniscus damage, other reasons for an increased risk of reduced muscle strength and loss of pre-injury activity levels may be related to inadequate fracture reduction and fixation, which does not allow early functional rehabilitation [4, 6, 9, 10, 40]. In this context, various strategies have been proposed to achieve absolute stability and interfragmentary compression of the avulsed tibial tubercle, with screw fixation representing the most common internal fixation method [4]. However, the current literature lacks reliable evidence regarding proper fixation of Ogden fractures with epiphyseal and/or intra-articular involvement, which are frequently observed in clinical practice [4, 5, 10, 13, 25].

To the best of our knowledge, this is the first study evaluating the biomechanical performance of different fixation techniques in tibial tubercle avulsion fractures. In tibial tubercle osteotomy (TTO), recent biomechanical studies have shown that isolated screw fixation of the tibial tubercle counteracts the vertical shear forces across the osteotomy site [35, 36]. Nurmi et al. [35] have demonstrated in a human cadaveric bone model that isolated screw fixation of the TTO with two 3.5 mm metal screws resisted higher failure loads (1163 N) than fixation with two 3.5 mm bioabsorbable screws (724 N). In the present study, isolated screw fixation provided equivalent biomechanical stability for fixation of the Ogden type IIIA and Ogden type IV fragment with comparable cycles to failure and failure loads. However, the failure loads were remarkably lower compared to the aforementioned study by Nurmi et al. [35], which might be due to the different shape of the Ogden fragment and the TTO. The fragments following TTO are smaller than Ogden IIIA/IV fragments and provide less bony contact surface, but relatively higher interfragmentary compression and friction leading to higher fixation strength of the isolated screw osteosynthesis.

However, in these Ogden type IIIA/IV fractures, the stability of the isolated apophyseal screw fixation might be improved, when augmentation with epiphyseal screws or locking plates is performed. In 2013, Warner et al. [36] have shown in a in a human cadaveric bone model that the use of an additional screw in anteromedialization TTO further improved the ultimate fixation strength. Using a comparable testing setup and loading protocol as Nurmi et al. [35], screw osteosynthesis of the TTO with three 3.5 mm cortical screws exhibited a mean maximum failure load of 1360 N, which is 15% higher than the proposed two-screw construct by Nurmi et al. [35] mentioned above. Even though the augmented constructs in the present study - both the epiphyseal screws and the medial plate - did not achieve failure loads comparable to the aforementioned studies [35, 36], augmented apophyseal screw fixation still resulted in a significant reduction in fracture site displacement and significantly improved failure loads. However, the fact that augmentation of a two-screw construct provides a significant stabilizing effect supports the concept of augmented fixation in tibial tubercle avulsion fractures with epiphyseal and/or intra-articular involvement. Thus, if these coronal plane fractures of the tibial tubercle are not treated with an appropriate fixation strategy that neutralizes the vertical tensile forces of the extensor mechanism, the osteosynthesis is likely to fail.

The results of the present study are of clinical relevance considering that the extensor mechanism and thus the osteosynthesis are subjected to high forces and torques during flexion-extension cycles of the knee [22–24, 41]. In fact, the average maximum quadriceps force exerted to the tibial tubercle during knee extension in normal gait is estimated to be approximately 300 N, so that this value can be considered as the estimated fixation strength required for safe fixation of the avulsed tibial tubercle [23, 41]. In the present study, isolated fixation of the tibial tubercle with two screws did not exceed the estimated maximum quadriceps pull-out forces at the tibial tubercle for both Ogden type IIIA and IV fractures (148 N and 127 N) and should therefore not be advocated for treatment of apoepiphyseal Ogden fractures from a biomechanical point of view. For Ogden IIIA fractures, additional epiphyseal fixation with two screws or a medial plate provided the required fixation strength (296 N and 304 N) and neutralized the excessive extensor mechanism forces at the tibial tubercle. However, symptomatic hardware with residual pain is the most common complication after surgical treatment of Ogden fractures and the main reason for reoperations [3–5, 9]. Therefore, with comparable biomechanical stability, augmentation of apophyseal screw osteosynthesis by two additional epiphyseal screws is preferable to a prominent medial plate in Ogden IIIA fractures, as this technique might not endanger the physis and might not further increase the risk of complications. In addition, the epiphyseal screws provide interfragmentary compression of the coronal split fracture at the tibial plateau to reduce intra-articular step-offs and gaps, which have been shown to improve patient-reported outcomes and to reduce the risk of osteoarthritis in tibial plateau fractures [31, 32, 42]. In contrast, augmented epiphyseal screw fixation might not be suitable in Ogden IV fractures, as the entire epiphysis is avulsed. To avoid damage to the physis, augmentation by a physis-spanning medial plate fixation appears to be more appropriate. For this augmented construct, improved fixation strength has been shown compared to isolated apophyseal screw osteosynthesis (158 N vs. 127 N), but without neutralizing the excessive extensor mechanism forces at the tibial tubercle. Therefore, despite augmented fixation, cautious rehabilitation with prolonged partial weight bearing and gradual increase in range of motion might be necessary in Ogden IV fractures to avoid vertical dislocation of the tibial tubercle and thus subsequent extensor mechanism insufficiency. However, the plate should be removed early after fracture healing to avoid permanent hemiphysiodesis with subsequent varus deformity [6, 8]. In contrast, augmented apophyseal screw fixation of Ogden IIIA fractures with additional epiphyseal screws or a medial plate neutralized the extensor mechanism forces at the tibial tubercle. Therefore, these fractures might be treated with early functional rehabilitation with unrestricted range of motion immediately postoperatively and a short period of partial weight bearing, to avoid knee joint stiffness and quadriceps atrophy. Moreover, high-grade apoepiphyseal Ogden fractures (Type III– V) are often present with severe soft tissue swelling due to the high-energy injury mechanism, so in these compromised soft tissue situations, minimally invasive percutaneous apoepiphyseal screw osteosynthesis should be performed for Ogden IIIA fractures. In Ogden IV fractures, however, plate augmentation is more stable than screw fixation alone and should only be used when the soft tissue envelope allows a more extensive surgical incision.

The present study has several limitations that should be considered before interpreting the results. First, cadaveric knee specimens of older age (age 76.0 ± 7.3 years) were used, which might not necessarily reflect the bone a quality of adolescent patients suffering from Ogden fractures [4, 10]. Nonetheless, the proximal tibiae were assigned to the groups on the basis of BMD to ensure biomechanical testing in non-osteoporotic specimens and comparability between the different test conditions [43]. Second, the used fracture model represents only a simplification of the partly inhomogeneous fracture patterns of Ogden fractures. Furthermore, the fractures were simulated by osteotomies, which might not necessarily reflect the epiphyseolysis and cartilaginous physis in adolescent patients. Nevertheless, the used fracture model is characterized by a high reproducibility, which enables reliable conclusions regarding the stability of augmented constructs and thus the development of a surgical strategy based on the fracture morphology. Last, the biomechanical testing simulated forces acting at time zero, where biological factors and bone healing were not taken into account. Furthermore, unidirectional testing was chosen in order to simulate a worst-case scenario, which may not mimic the forces acting in vivo during early rehabilitation.

## Conclusion

Augmented apophyseal screw fixation of apoepiphyseal tibial tubercle avulsion fractures provides greater biomechanical stability than isolated apophyseal screw fixation. Regardless of fixation technique, Ogden type IV fractures are more unstable than Ogden type IIIA fractures, so an individualized treatment strategy based on fracture morphology is crucial. In case of an Ogden type IIIA or Ogden type IV fracture, surgeons should consider adding epiphyseal screws or a medial plate osteosynthesis to apophyseal screw fixation to best neutralize forces of the extensor mechanism, as long as the often compromised soft tissue envelope can tolerate greater surgical invasiveness.

## Data Availability

No datasets were generated or analysed during the current study.

## Abbreviations

SD: Standard deviation.
BMD: Bone mineral density.
CT: Computed tomography.
LCP: Locking compression plate.
PMMA: Polymethylmethacrylate.
TTO: Tibial tubercle osteotomy.

## Declarations

### Level of evidence

There is no level of evidence as this study was an experimental laboratory study.
